# Temporal Trends in the Management and Mortality Associated With Klebsiella pneumoniae Carbapenemase-Producing Enterobacterales: A Cohort Study

**DOI:** 10.7759/cureus.81902

**Published:** 2025-04-08

**Authors:** Gonçalo Pinto, Francisca Bartilotti Matos, Ana Gorgulho, Tiago Teixeira, Rosa Oliveira, Vera Gomes, Nuno Vieira, Leila Ramdani, Gabriela Abreu, Luís Malheiro

**Affiliations:** 1 Department of Medicine, Faculdade de Medicina da Universidade do Porto, Porto, PRT; 2 Department of Infectious Diseases, Unidade Local de Saúde Gaia/Espinho, Vila Nova de Gaia, PRT; 3 Local Unit of the Infection Prevention and Control and Antimicrobial Resistance Program, Unidade Local de Saúde Gaia/Espinho, Vila Nova de Gaia, PRT; 4 Department of Microbiology, Unidade Local de Saúde Gaia/Espinho, Vila Nova de Gaia, PRT; 5 Department of Infectious Diseases, Unidade Local de Saúde Gaia/Espinho, Vila Nova De Gaia, PRT; 6 Department of Infectious Diseases, Departamento de Ciências Médicas da Universidade de Aveiro, Aveiro, PRT

**Keywords:** antimicrobial resistance, antimicrobial stewardship, beta-lactamase inhibitors, carbapenem-resistant enterobacterales, klebsiella pneumoniae carbapenemase

## Abstract

Introduction

*Klebsiella pneumoniae* carbapenemase-producing Enterobacterales (KPC-CPE) are a significant cause of healthcare-associated infections, characterized by high-level resistance to beta-lactam antibiotics and limited therapeutic options. This study aimed to analyze the epidemiological trends, clinical management, and mortality associated with KPC-CPE infections over a decade, highlighting variations in incidence and treatment patterns during and after the COVID-19 pandemic.

Methods

A retrospective, single-center cohort study was conducted at a tertiary Portuguese hospital, analyzing data from August 2015 to June 2024. Patients with microbiologically confirmed KPC-CPE infections were included in this study. Epidemiological, clinical, and therapeutic data were extracted and analyzed using descriptive statistics and logistic regression to identify risk factors for mortality.

Results

Among 6,259 patients with KPC-CPE isolates, 483 (7.7%) developed infections. Infection rates peaked in 2016 and 2023, with a decline during the COVID-19 pandemic. The 30-day mortality rate was 28%, with bloodstream infections (BSIs) (odds ratio {OR}=1.64, p=0.028) and admission to the intensive care unit (ICU) significantly associated with increased mortality. Urinary tract infections (UTIs) were significantly more frequent in survivors (p=0.001). A shift from combination therapy to monotherapy, particularly with ceftazidime-avibactam (CZA), was observed, aligning with international guidelines. Patients who did not receive adequate antibiotic treatment had significantly higher mortality (OR=6.36, p<0.001). Monotherapy with aminoglycosides, ceftazidime-avibactam, tigecycline, co-trimoxazole (SXT), or fluoroquinolones was more common in survivors. Conversely, combination therapies involving high-dose meropenem (HD-MEM) or aminoglycosides were more common among non-survivors. Mortality was exceptionally high in 2019 and 2020, with no single explanatory factor identified.

Conclusion

Our study findings highlight the importance of rigorous infection control measures, the optimization of antimicrobial therapy, and the continuous surveillance of antimicrobial resistance. The growing reliance on monotherapy underscores the necessity of antimicrobial stewardship programs to prevent the development of resistance. Additional multicenter studies are needed to optimize therapeutic strategies and improve patient outcomes.

## Introduction

Infections caused by carbapenemase-producing Enterobacterales(CPE), particularly those producing *Klebsiella pneumoniae* carbapenemase (KPC), represent a significant global health challenge [1-6]. KPC-producing Enterobacterales(KPC-CPE) are difficult-to-treat gram-negative bacilli that are resistant to numerous antibiotics, especially beta-lactams. These bacteria are frequently implicated in healthcare-associated infections, particularly affecting the urinary tract, respiratory system, and bloodstream, being associated with high morbidity and mortality, usually exacerbated by patients’ underlying comorbidities [1,7,8].

Beta-lactam resistance considerably limits the available therapeutic options, often driving clinicians to prescribe drugs with higher toxicity, such as polymyxins and aminoglycosides [1,7,9-11]. Treatment approaches have evolved significantly; colistin, initially recommended, was later associated with side effects and resistance. Consequently, clinicians adopted combination antimicrobial therapies by supplementing colistin with fosfomycin, aminoglycosides, or tigecycline, thereby mitigating resistance risks and enhancing patient outcomes [7,9-12].

Recently, new beta-lactam/beta-lactamase inhibitor combinations, including ceftazidime-avibactam (CZA), meropenem-vaborbactam, and imipenem-relebactam, have shown promise for treating infections caused by KPC-CPE [2,9,13]. Despite these developments, KPC-CPE infections continue to impose substantial economic burdens on healthcare systems worldwide [1,14].

In Portugal, a steady increase in KPC-CPE infections and nosocomial outbreaks highlights ongoing challenges, such as limited effective treatments, extended hospital stays, and escalating healthcare costs [1,14].

The impact of evolving antimicrobial therapy and infection control measures on clinical outcomes remains unclear. Therefore, this study aimed to analyze the temporal trends in the clinical management and mortality associated with KPC-CPE infections, providing critical insights to inform clinical strategies and public health policies. Specifically, we examined the evolution of treatment practices, including the use of different antimicrobial regimens, and their correlation with mortality trends.

## Materials and methods

This retrospective, single-center cohort study was conducted at the Unidade Local de Saúde Gaia/Espinho (ULSGE), a tertiary care hospital in Portugal. Data were collected from August 15, 2015, following the identification of the first carbapenemase-producing microorganism, to June 2024. The study was approved by the Research Ethics Committee of the Unidade Local de Saúde Gaia/Espinho (approval number: 184/2024-1), and a waiver for informed consent was granted.

Study population

The study included all adult patients with microbiologically confirmed KPC-CPE isolates during the study period. From these, only patients with infection were selected for analysis. Infection was defined by the attending clinician at the time of patient evaluation. This determination required the isolation of KPC-CPE from clinical specimens plus clinical evidence of infection. Such evidence included systemic signs (e.g., fever, chills, hypotension, and tachypnea), foci-specific symptoms (e.g., cough, sputum production, and pleuritic chest pain in respiratory infections; dysuria, urinary frequency, and flank pain in urinary tract infections {UTIs}; or abdominal tenderness in intra-abdominal infections), a host inflammatory response (as indicated by elevated C-reactive protein and procalcitonin levels), or the initiation of targeted antimicrobial therapy. Colonization was defined as the isolation of KPC-CPE from either a rectal swab or biological sample without the associated clinical symptoms, inflammatory response, or treatment indication.

Carbapenem-resistant Enterobacterales(CRE) isolated from rectal swabs, as part of the hospital’s screening policy, were identified using selective chromogenic agar plates. Colonies suggestive of CRE were then subjected to confirmatory testing for the most common carbapenemase enzyme, using either phenotypic immunochromatographic methods or polymerase chain reaction (PCR) assays.

CRE isolates from other biological samples underwent identification and antimicrobial susceptibility testing (AST) using matrix-assisted laser desorption/ionization time-of-flight (MALDI-TOF) mass spectrometry (VITEK® MS, bioMérieux, Marcy-l’Étoile, France) and turbidimetry (VITEK® 2, bioMérieux, Marcy-l’Étoile, France), respectively. AST results were interpreted according to the guidelines of the European Committee on Antimicrobial Susceptibility Testing (EUCAST) [15]. In cases of carbapenem resistance, KPC production was determined using phenotypic immunochromatographic or PCR assays.

Data collection

Information was extracted from the institutional microbiological surveillance software, which provided standardized access to clinical and microbiological records. We implemented rigorous data collection protocols and quality control measures to maximize data completeness and ensure that missing data remained minimal. For infected patients, the collected variables included age, sex, semester and year of infection, hospital ward (medical, surgical, or ICU), the type of carbapenem-resistant microorganism, and the biological sample in which the microorganism was isolated. We also recorded the interval between colonization and infection and the AST patterns. Additional data included the type of infection, classified according to the European Centre for Disease Prevention and Control (ECDC) case definition for infection surveillance; the presence of bacterial coinfection; administered antibiotics (including the use of combination therapy); 30-day all-cause mortality; 30-day infection-related mortality; and total length of hospital stay [16]. Adequate antibiotic therapy was defined through expert clinical judgment in accordance with our antimicrobial stewardship program as treatment with agents that demonstrate in vitro activity against the etiologic pathogens (as determined by standardized AST protocols), tailored to the source of infection and administered in line with current clinical guidelines using the correct dose, route, and duration.

Statistical analysis

Based on an anticipated mortality rate of 37%-43% for infections caused by KPC-producing microorganisms, a sample size of 359-379 participants was calculated to ensure that the true rate was estimated with a ±5% margin of error at a 95% confidence level [17]. Descriptive statistics were used to summarize the baseline characteristics. Continuous variables are expressed as mean±standard deviation (SD) for normally distributed data and as median with interquartile range (IQR) for non-normally distributed data. Comparisons between survivors and non-survivors were performed using chi-square tests for categorical variables and Student’s t-tests or Mann-Whitney U tests for continuous variables, as appropriate. For contingency table analyses, p-values are reported based on either the chi-square test or Fisher’s exact test (when appropriate), along with the degrees of freedom (df) and an effect size measure, using the phi coefficient (φ) for 2×2 tables and Cramér’s V for larger tables. A logistic regression model with effect (deviation) coding was used to evaluate the odds of infection across years and locations so that each category’s coefficient reflects its deviation from the grand mean rather than a single reference group. Multicollinearity among treatment variables and severity indicators (e.g., intensive care unit {ICU} status) was checked via variance inflation factors, applying a threshold of 10 to identify severe collinearity. Results are presented as odds ratios (OR) with 95% confidence intervals (CI). Analyses were conducted using IBM SPSS Statistics version 27.0 (IBM Corp., Armonk, NY), and p<0.05 was considered statistically significant.

## Results

Epidemiological trends and demographics

**Figure 1:**
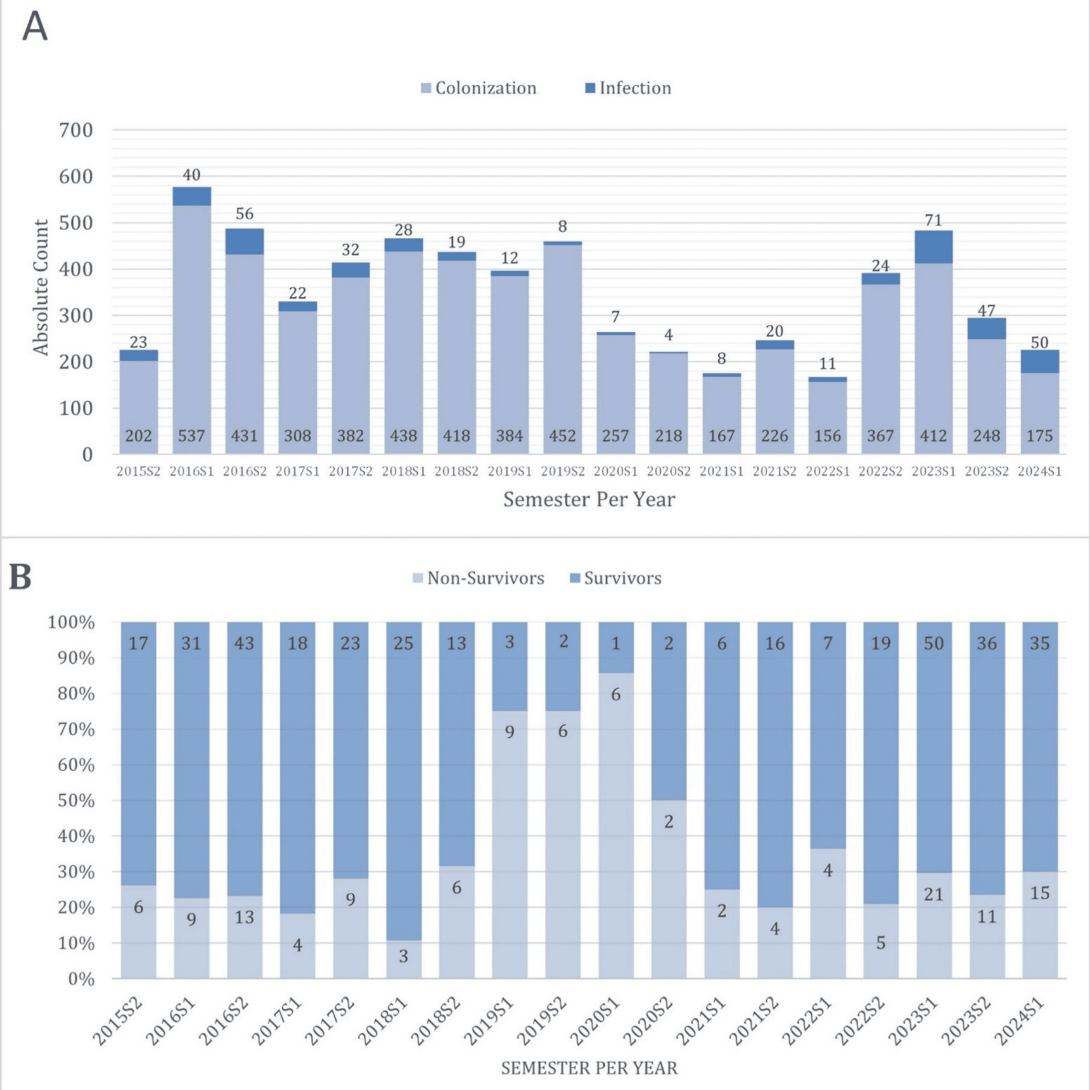
Absolute count of colonized and infected cases per semester and year (a) and the percentage of survivors and non-survivors among infected patients per semester and year (B)

Between August 7, 2015, and June 30, 2024, 6,259 patients were identified as having KPC-CPE isolated from their biological samples. Figure 1A shows the number of colonization events and infections each year. The annual incidence showed significant variation, initially increasing from 225 patients (3.6%) in 2015 to a peak of 1,064 in 2016. A marked decrease occurred during the COVID-19 pandemic, with 486 (7.7%) and 421 (6.6%) patients identified in 2020 and 2021, respectively. A resurgence was observed in 2023, with 776 patients (12.4%).

A total of 483 KPC-CPE infections were recorded, representing 7.7% of all identified cases. Most infected patients were men (273, 56.5%) compared to women (210, 43.5%). We assessed the extent of missing data for all variables and found that the proportion of missing information was minimal for most parameters.

Table 1 describes the characteristics of the infected patients. The highest annual infection rate was observed in 2023 (118/776, 15.2%), followed by 2016 (96/1,064, 9.0%). Conversely, the lowest infection rates were recorded in 2019 and 2020 (2.3% each year), simultaneously with the COVID-19 pandemic. Surgical wards had the highest proportion of infections (154 cases, 31.9%), whereas the intensive care unit (ICU) had the highest odds ratio (OR) for infection (2.31, p<0.001, 95% CI: 1.80-2.97). In contrast, the surgical and medical wards had mean odds of infection of 79.5% (p=0.007, 95% CI: 0.67-0.94) and 54.5% (p<0.001, 95% CI: 0.30-0.91). Compared to the overall mean odds for infection, 2015 had 1.81 times the mean (p=0.004, 95% CI: 1.21-2.69), 2016 had 1.49 times (p<0.001, 95% CI: 1.19-1.86), and 2023 had 2.39 times (p<0.001, 95% CI: 1.91-2.97). In contrast, 2019 showed only 28.7% of the mean odds (p<0.001, 95% CI: 0.18-0.45), and 2020 had 30.4% (p<0.001, 95% CI: 0.17-0.54). There were no statistical differences between the years and locations.

**Table 1:** Characteristics of patients with KPC-CPE infection CSF, cerebrospinal fluid; CVC, central venous catheter; ICU, intensive care unit; LRTI, lower respiratory tract infection (pneumonia and other lower respiratory tract infections); SSTI, skin and soft tissue infection that includes cellulitis and soft tissue and wound infections; UTI, urinary tract infection; V, Cramér’s V; χ², chi-square; df, degrees of freedom; φ, phi coefficient; KPC-CPE, *Klebsiella pneumoniae* carbapenemase-producing Enterobacterales

	Total (n=483)	Non-survivor (n=135)	Survivor (n=348)	χ²(df), p-value, φ/V
Sex				χ²(1)=1.65, p=0.165, φ=-0.06
Male, n (%)	273 (56.52)	83 (61.48)	190 (54.60)	
Female, n (%)	210 (43.48)	52 (38.52)	158 (45.40)	
Year of infection				χ²(9)=32.89, p=0.001, φ=0.26
2015, n (%)	23 (4.76)	6 (4.44)	17 (4.89)	
2016, n (%)	96 (19.88)	22 (16.30)	74 (21.26)	
2017, n (%)	54 (11.18)	13 (9.63)	41 (11.78)	
2018, n (%)	47 (9.73)	9 (6.67)	38 (10.92)	
2019, n (%)	20 (4.14)	15 (11.11)	5 (1.44)	
2020, n (%)	11 (2.28)	8 (5.93)	3 (0.86)	
2021, n (%)	28 (5.80)	6 (4.44)	22 (6.32)	
2022, n (%)	35 (7.25)	9 (6.67)	26 (7.47)	
2023, n (%)	118 (24.43)	32 (23.70)	86 (24.71)	
2024, n (%)	51 (10.56)	15 (11.11)	36 (10.34)	
Age (in decades)				χ²(7)=5.74, p=0.588, V=0.11
18-29, n (%)	4 (0.83)	1 (0.74)	3 (0.86)	
33-39, n (%)	8 (1.66)	1 (0.74)	7 (2.01)	
40-49, n (%)	19 (3.93)	3 (2.22)	16 (4.60)	
50-59, n (%)	73 (15.11)	17 (12.59)	56 (16.09)	
60-69, n (%)	103 (21.33)	35 (25.93)	68 (19.54)	
70-79, n (%)	152 (31.47)	41 (30.37)	111 (31.90)	
80-89, n (%)	107 (22.15)	31 (22.96)	76 (21.84)	
>90, n (%)	17 (3.52)	6 (4.44)	11 (3.16)	
Location at infection diagnosis				χ²(3)=11.024, p=0.012, V=0.15
ICU, n (%)	100 (20.70)	40 (29.63)	60 (17.24)	
Medical ward, n (%)	144 (29.81)	31 (22.96)	113 (32.47)	
Surgical ward, n (%)	154 (31.88)	44 (32.59)	110 (31.61)	
Emergency, n (%)	85 (17.60)	20 (14.81)	65 (18.68)	
Microorganism				
*Klebsiella pneumoniae*, n (%)	455 (94.20)	131 (97.04)	324 (93.10)	χ²(1)=3.89, p=0.046, φ=0.90
*E. coli*, n (%)	32 (6.63)	6 (4.44)	26 (7.47)	χ²(1)=1.38, p=0.230, φ=-0.05
Others, n (%)	3 (0.62)	0 (0.00)	3 (0.86)	χ²(1)=0.39, p=0.999, φ=-0.3
Biological sample				
Blood, n (%)	99 (20.50)	36 (26.67)	63 (18.10)	χ²(1)=3.59, p=0.036, φ=0.09
CVC, n (%)	3 (0.62)	0 (0.00)	3 (0.86)	χ²(1)=1.16, p=0.279, φ=-0.05
Pus/exudate, n (%)	66 (13.66)	12 (8.89)	54 (15.52)	χ²(1)=3.49, p=0.057, φ=-0.9
Peritoneal fluid, n (%)	31 (6.42)	12 (8.89)	19 (5.46)	χ²(1)=1.99, p=0.168, φ=0.06
Bronchoalveolar lavage, n (%)	14 (2.90)	6 (4.44)	8 (2.30)	χ²(1)=1.65, p=0.200, φ=0.06
Sputum/tracheal aspirate, n (%)	58 (12.01)	23 (17.04)	35 (10.06)	χ²(1)=4.67, p=0.034, φ=0.09
Pleural fluid, n (%)	9 (1.86)	3 (2.22)	6 (1.72)	χ²(1)=0.14, p=0.716, φ=0.02
Urine, n (%)	192 (39.75)	41 (30.37)	151 (43.39)	χ²(1)=6.49, p=0.009, φ=-0.12
Biopsy, n (%)	11 (2.28)	3 (2.22)	8 (2.30)	χ²(1)=0.01, p=0.960, φ=-0.01
CSF, n (%)	2 (0.41)	1 (0.74)	1 (0.29)	χ²(1)=0.49, p=0.486, φ=0.03
Synovial fluid/bone, n (%)	2 (0.41)	0 (0.00)	2 (0.57)	χ²(1)=0.77, p=1.000, φ=-0.04
Source of infection				
LRTI, n (%)	81 (16.77)	30 (22.22)	51 (14.66)	χ²(1)=4.19, p=0.046, φ=0.09
Surgical site, n (%)	47 (9.73)	12 (8.89)	35 (10.06)	χ²(1)=0.48, p=0.697, φ=-0.3
Bloodstream, n (%)	82 (16.98)	30 (22.22)	52 (14.94)	χ²(1)=3.39, p=0.056, φ=0.09
UTI, n (%)	188 (38.92)	37 (27.41)	151 (43.39)	χ²(1)=9.98, p=0.001, φ=-0.14
SSTI, n (%)	24 (4.97)	4 (2.96)	20 (5.75)	χ²(1)=1.55, p=0.206, φ=-0.06
Intra-abdominal, n (%)	37 (7.66)	13 (9.63)	24 (6.90)	χ²(1)=1.09, p=0.311, φ=0.05
Reproductive tract infection, n (%)	1 (0.21)	0 (0.00)	1 (0.29)	χ²(1)=0.39, p=1.000, φ=-0.03
Bone and joint infection, n (%)	5 (1.04)	0 (0.00)	5 (1.44)	χ²(1)=1.94, p=0.329, φ=-0.063
Central nervous system infection, n (%)	2 (0.41)	1 (0.74)	1 (0.29)	χ²(1)=0.49, p=0.486, φ=0.03
Systemic infection/sepsis, n (%)	16 (3.31)	8 (5.93)	8 (2.30)	χ²(1)=4.09, p=0.084, φ=0.09
Received adequate antibiotic therapy, n (%)	423 (87.58)	100 (71.43)	323 (94.17)	χ²(1)=30.56, p=0.001, φ=-0.25
Other microorganisms identified	40 (8.28)	11 (7.86)	29 (8.45)	χ²(1)=0.01, p=0.947, φ=-0.01
Time since colonization to infection				χ²(3)=0.81, p=0.832, φ=0.04
0-7 days, n (%)	224 (46.38)	62 (44.29)	162 (47.23)	
8-28 days, n (%)	112 (23.19)	29 (20.71)	83 (24.20)	
29-365 days, n (%)	121 (25.05)	35 (25.00)	86 (25.07)	
>365 days, n (%)	26 (5.38)	9 (6.43)	17 (4.96)	

Clinical and infection-related characteristics

Urine was the most common biological sample from which KPC-CPE was isolated (192, 39.8%), followed by blood (99, 20.5%) and lower respiratory tract samples (58, 12.0%). The most frequent infection sources were urinary tract infections (UTIs) (188, 38.9%) and bloodstream infections (BSIs) (82, 17.0%). Lower respiratory tract infections (LRTIs) accounted for 81 cases (16.8%), and surgical site infections were reported in 47 cases (9.7%). Surgical site and intra-abdominal infections were significantly more common in the surgical ward (p<0.001). UTI predominated in medical wards and emergency settings (p<0.001), skin and soft tissue infections (SSTI) were mostly diagnosed in the medical and surgical wards (p=0.005), whereas LRTIs were most common in the ICU (56.8%, p<0.001).

Antibiotic therapy data showed that 423 (87.6%) patients received adequate directed antibiotic therapy, whereas 60 (12.4%) did not. Table 2 describes the treatments used in our cohort. Monotherapy was the predominant treatment approach (62.3%), primarily using aminoglycosides (17.8%) and ceftazidime-avibactam (CZA) (17.6%). Combination therapy was employed in 122 cases (25.3%), mostly with two-drug regimens. The commonly used combinations included aminoglycosides with tigecycline (7.3%), high-dose meropenem (HD-MEM) with aminoglycosides (3.5%), and tigecycline with colistin (3.1%).

**Table 2:** Treatment description of patients with KPC-CPE infection with univariate and multivariate analysis for 30-day mortality ^1^Other antimicrobials included nitrofurantoin (n=2), cefepime (n=3), amoxicillin/clavulanate (n=1), and meropenem-vaborbactam (n=1) ^2^Thecombination HD-MEM+others includes FOS (n=3, two survivors), TGC (n=2, one survivor), COL (n=3, two survivors), quinolone (n=3, two survivors), and SXT (n=3, one survivor) ^3^Aminoglycoside+others includes SXT (n=4, four survivors) and quinolone (n=2, two survivors) ^4^Other two-drug combination includes FOS+SXT (n=1, one survivor), COL+FOS (n=1, zero survivors), and SXT+quinolone (n=1, one survivor) ^5^>2-drug combinations: aminoglycoside+FOS+TGC (n=2, two survivors), aminoglycoside+FOS+COL (n=2, two survivors), HD-MEM+FOS+quinolone (n=1, one survivor), and aminoglycoside+FOS+TGC+COL (n=1, zero survivors) ^6^Variables present in the formula but not shown in the table due to nonsignificant p-value: age, sex, location at infection diagnosis, source of infection, and other microorganisms identified AG, aminoglycoside; COL, colistin; CZA, ceftazidime/avibactam; FOS, fosfomycin; HD-MEM, high-dose meropenem; SXT, co-trimoxazole; TGC, tigecycline; V, Cramér’s V; χ², chi-square; df, degrees of freedom; φ, phi coefficient; OR, odds ratio; CI, confidence interval; KPC-CPE, *Klebsiella pneumoniae *carbapenemase-producing Enterobacterales

	Univariate analysis				Multivariate analysis^6^	
	Total (n=483)	Non-survivor (n=140)	Survivor (n=343)	χ²(df), p-value, φ/V	P-value	OR (95% CI)
Monotherapy, n (%)	301 (71.16)	67 (63.81)	234 (72.45)	χ²(1)=13.48, p=0.500, φ=-0.17	-	-
AG, n (%)	86 (20.33)	14 (13.33)	72 (22.29)	χ²(1)=9.08, p=0.003, φ=-0.14	<0.001	0.233 (0.108-0.502)
CZA, n (%)	85 (20.09)	24 (22.86)	61 (18.89)	χ²(1)=0.01, p=0.911, φ=0.01	0.001	0.294 (0.138-0.625)
HD-MEM, n (%)	21 (4.96)	6 (5.71)	15 (4.64)	χ²(1)=1.149, p=0.284, φ=0.05	0.042	0.315 (0.103-0.960)
FOS, n (%)	25 (5.91)	8 (7.62)	17 (5.26)	χ²(1)=0.09, p=0.771, φ=-0.01	0.759	0.859 (0.324-2.276)
TGC, n (%)	21 (4.96)	5 (4.76)	16 (4.95)	χ²(1)=0.18, p=0.673, φ=-019	0.026	0.280 (0.091-0.862)
COL, n (%)	13 (3.07)	4 (3.81)	9 (2.79)	χ²(1)=0.97, p=0.324, φ=0.05	0.165	0.403 (0.111-1.454)
SXT, n (%)	24 (5.67)	3 (2.86)	21 (6.50)	χ²(1)=2.80, p=0.094, φ=-0.78	0.037	0.286 (0.088-0.928)
Quinolone, n (%)	19 (4.49)	2 (1.90)	17 (5.26)	χ²(1)=3.26, p=0.071, φ=-0.8	0.002	0.116 (0.030-0.458)
Other antimicrobials^1^, n (%)	7 (1.65)	1 (0.95)	6 (1.86)	χ²(1)=0.25, p=0.456, φ=0.05	0.899	0.865 (0.564-7.456)
Combination therapy, n (%)	122 (28.84)	33 (31.43)	89 (27.55)	χ²(1)=0.04, p=0.586, φ=-0.01	-	-
Two-drug combinations, n (%)	116 (27.42)	32 (30.48)	84 (26.01)	χ²(1)=0.01, p=0.965, φ=-0.01	-	-
HD-MEM+AG, n (%)	17 (4.02)	6 (5.71)	11 (3.41)	χ²(1)=0.50, p=0.479, φ=0.32	0.036	5.829 (1.120-30.332)
HD-MEM+others^2^, n (%)	14 (3.31)	6 (5.71)	8 (2.48)	χ²(1)=1.64, p=0.200, φ=0.06	0.035	5.970 (1.130-31.543)
AG+TGC, n (%)	35 (8.27)	9 (8.57)	26 (8.05)	χ²(1)=0.08, p=0.781, φ=-0.01	0.045	4.175 (1.030-16.925)
AG+FOS, n (%)	10 (2.36)	0 (0.00)	10 (3.10)	χ²(1)=3.92, p=0.069, φ=-0.9	0.999	0.000 (0.000-0.000)
AG+COL, n (%)	9 (2.13)	3 (2.86)	6 (1.86)	χ²(1)=0.71, p=0.713, φ=0.02	0.127	4.785 (0.642-35.662)
AG+others^3^, n (%)	6 (1.42)	0 (0.00)	6 (1.86)	χ²(1)=2.33, p=0.193, φ=-0.07	0.999	0.000 (0.000-0.000)
TGC+COL, n (%)	16 (3.78)	6 (5.71)	10 (3.10)	χ²(1)=0.79, p=0.398, φ=0.04	0.135	4.215 (0.638-27.832)
TGC+FOS, n (%)	6 (1.42)	1 (0.95)	5 (1.55)	χ²(1)=0.37, p=1.000, φ=-0.03	0.669	0.570 (0.044-7.463)
Other two-drug combinations^4^, n (%)	3 (0.71)	1 (0.95)	2 (0.62)	χ²(1)=0.05, p=1.000, φ=0.01	0.807	1.552 (0.046-52.480)
>2 drug combinations^5^, n (%)	6 (1.42)	1 (0.95)	5 (1.55)	χ²(1)=0.372, p=1.000, φ=-0.03	0.159	7.920 (0.443-141.427)

**Figure 2:**
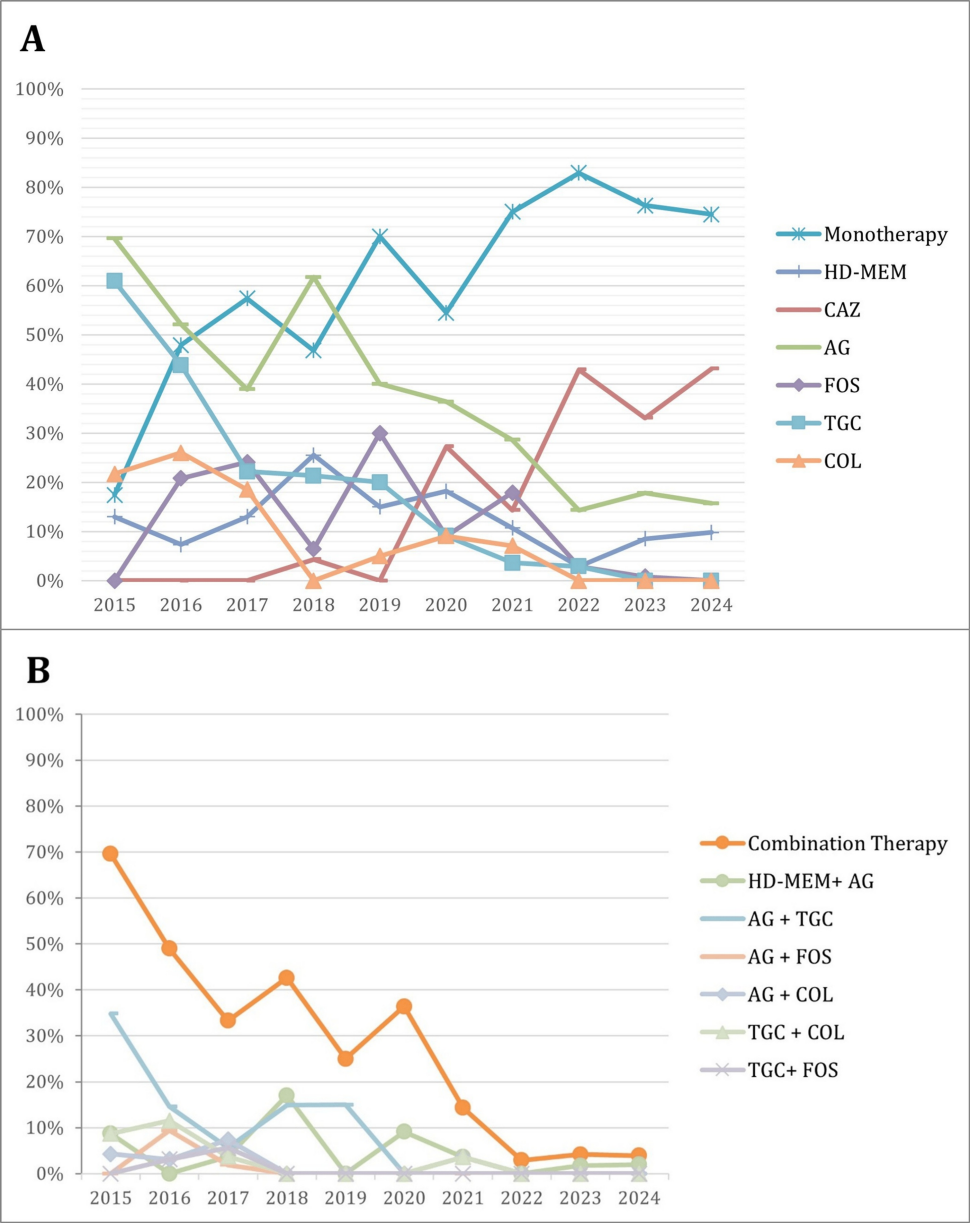
Antibiotic therapy in patients with KPC-CPE infections during the study period: (A) monotherapy and (B) combined therapy AG, aminoglycoside; COL, colistin; CZA, ceftazidime/avibactam; FOS, fosfomycin; HD-MEM, high-dose meropenem; SXT, co-trimoxazole; TGC, tigecycline; KPC-CPE, *Klebsiella pneumoniae* carbapenemase-producing Enterobacterales

Throughout the study period, monotherapy usage significantly increased from 17.4% in 2015 to 74.5% in 2024 (p<0.001), whereas combination therapy decreased from 69.6% to 3.9% (p<0.001) (Figure 2A and Figure 2B, respectively). Aminoglycoside usage declined after 2018, and fosfomycin utilization peaked in 2019 (30%) before declining sharply.

Mortality analysis

Figure 1B shows the mortality rate over the study period. A comparison between survivors and non-survivors is presented in Table 1 and Table 2. The overall 30-day mortality rate was 28% (135 patients). Mortality was significantly higher in ICU admissions (29.6% in non-survivors versus 17.2% in survivors, p=0.012), BSI (22.2% in non-survivors versus 14.9% in survivors, p=0.036), and LRTI (22.2% in non-survivors versus 14.7% in survivors, p=0.046). Conversely, UTIs were significantly more frequent among survivors (43.4% versus 27.4%, p=0.001).

Patients who did not receive adequate antibiotic therapy exhibited significantly higher odds for mortality (OR: 6.36, p<0.001, 95% CI: 3.40-11.90). No statistically significant differences in mortality were noted between the monotherapy and combination therapy groups in the univariate analysis.

In the multivariate analysis, considering all possible confounders, several antimicrobial regimens emerged as statistically significant predictors of 30-day mortality (p<0.05). Monotherapy with CZA, high-dose meropenem (HD-MEM), aminoglycoside, tigecycline (TGC), co-trimoxazole (SXT), or fluoroquinolone was associated with reduced odds (OR range: 0.13-0.33) of 30-day mortality. In contrast, combination regimens involving HD-MEM plus an aminoglycoside (OR=6.72, 95% CI: 1.32-34.24, p=0.022) or HD-MEM plus another agent (OR=5.11, 95% CI: 0.98-26.61, p=0.053) showed elevated odds of mortality, although the latter was borderline significant. Aminoglycoside and TGC combinations also increased the odds (OR=4.12, 95% CI: 1.02-16.60, p=0.047). Other variables in the model, including specific infection sites, age, and year of infection, were not statistically significant. Multivariate analysis confirmed significantly increased mortality odds for infections occurring in 2019 and 2020 (OR=7.19 and p<0.001 and OR=7.20 and p=0.002, respectively) and among ICU patients (OR=1.64, p=0.028, 95% CI: 1.06-2.53). No additional factors fully explained the mortality spikes in 2019 and 2020.

## Discussion

This study assessed the epidemiological trends, clinical characteristics, and treatment outcomes associated with KPC-CPE infections over nearly a decade. Our findings highlight significant variations in infection incidence, with notable peaks observed in 2016 and 2023 and a marked decline during the initial years of the COVID-19 pandemic. The peak incidence in 2016 likely reflects improved detection efforts and surveillance policies; however, it could also indicate a true outbreak phenomenon following the initial identification. The observed reduction in KPC cases during the pandemic likely reflects changes in hospital admission patterns, stricter infection control practices, and adjustments in diagnostic laboratory workflows during this period, which is consistent with similar international findings [18]. The subsequent resurgence in 2023 and 2024 is a pattern already reported in post-pandemic surveillance studies and raises concerns about the long-term sustainability of infection control strategies implemented during the pandemic and a potential shift in local antimicrobial resistance patterns [19,20]. Furthermore, ICU patients had 2.32 times higher odds of infection, probably due to increased exposure to invasive procedures, mechanical ventilation, and prolonged hospital stays, emphasizing the need for targeted prevention strategies in high-risk groups.

The overall 30-day mortality rate of 28% aligns with that of previous studies, underscoring the substantial clinical burden associated with KPC-CPE infections [17]. Mortality showed significant variability across the years, with pronounced increases in 2019 and 2020. The higher mortality rate in 2020 likely resulted from healthcare disruptions during the COVID-19 pandemic, including altered clinical practices, reduced hospital capacity, and resource redistribution [18-20]. However, the mortality spike in 2019 is not fully explained by the available data, suggesting that unmeasured factors may have played a critical role. Prospective multicenter studies are needed to elucidate these unexplained fluctuations and further evaluate the impact of different clinical practices and infection control interventions on patient outcomes.

Consistent with previous literature, our data identified a significantly increased risk of mortality among ICU patients [8,17]. ICU admission is a known risk factor for mortality, attributable to increased exposure to invasive devices, prolonged hospitalization, and severe underlying clinical conditions. Our findings reinforce the need for targeted preventive strategies in the ICU, such as rigorous adherence to ventilator-associated pneumonia prevention bundles, including minimizing sedation, maintaining head-of-bed elevation, and performing regular oral care and subglottic suctioning.

BSIs and LRTIs were significantly associated with higher mortality rates, reflecting their severe clinical presentation and therapeutic challenges, particularly in critically ill patients. In contrast, UTIs showed significantly lower mortality, suggesting that the infection source influences patient prognosis. Given that UTIs were the most frequent infection type, predominantly occurring in medical wards, targeted interventions focusing on urinary catheter management, including timely catheter removal and adherence to aseptic insertion techniques, are essential for reducing these infections [21].

Our analysis revealed a notable shift from combination therapy to monotherapy over the study period, particularly following the introduction of CZA into the pharmacy formulary in 2019. This transition aligns with current international guidelines, which advocate for targeted monotherapy due to its comparable efficacy and improved safety profiles compared to broad-spectrum combinations [22,23]. Furthermore, institutional factors appear to have played a significant role; for example, a dedicated antimicrobial stewardship program for KPC-CPE treatment was initiated in 2021, likely influencing prescribing practices by promoting more targeted therapeutic strategies. However, the long-term efficacy of monotherapy remains uncertain due to the potential emergence of resistance. Indeed, we documented one instance of CZA resistance in KPC-CPE, emphasizing the need for continued surveillance of antimicrobial resistance.

Historically, combination therapies have been recommended because of their potential synergistic effects and reduced resistance emergence. However, recent clinical evidence has shown mixed results, questioning the consistent superiority of combination therapy over monotherapy [24,25]. After adjusting for other variables, our study observed significantly higher odds of mortality among patients receiving combination therapy, particularly combinations of HD-MEM or aminoglycosides. However, these findings may reflect biases related to treatment decisions and infection severity, as physicians may be more inclined to prescribe combination therapy for patients with more severe disease. In contrast, monotherapy, with the exception of fosfomycin and colistin, showed lower odds of mortality after adjusting for other relevant variables. Although our study was not powered to definitively confirm that monotherapy is superior to combination therapy, the promising results suggest that monotherapy, particularly with CZA, may be an effective treatment option for KPC-CPE infections. Notably, although findings have not been consistent across all studies, several other authors have reported similar outcomes with CZA monotherapy [26-28]. Despite variations in design and patient populations, these studies lend additional support to the use of monotherapy in appropriate clinical settings. While these observations are encouraging, further prospective research is needed to confirm these benefits and delineate the patient groups most likely to benefit from this approach. Future studies would benefit from incorporating more granular severity metrics, such as standardized severity scores, to better isolate the effects of treatment. Furthermore, the observation that patients treated with aminoglycosides, co-trimoxazole, and fluoroquinolones in monotherapy had lower odds of mortality suggests that some individuals, likely those with less severe conditions such as UTI, may benefit from CZA-sparing treatment strategies. Finally, although monotherapy with HD-MEM was associated with lower odds of mortality, this finding probably reflects patients with less severe KPC-producing *K. pneumoniae* infections with meropenem minimum inhibitory concentration (MIC) of ≤8 mg/L, in which this treatment may be recommended when other options are unavailable [22].

The impact of antibiotic therapy on survival was substantial, with patients who did not receive adequate therapy having 6.36 times the odds of mortality, reinforcing the critical role of timely and appropriate treatment. Additional research indicates that the time to adequate antibiotic administration is an independent risk factor for 30-day mortality among patients infected with KPC-producing *K. pneumoniae* [29]. These findings emphasize the critical importance of early and adequate antibiotic therapy, especially for patients in the ICU and those with BSI. Additionally, they suggested that targeted infection control and therapeutic strategies may help mitigate mortality risks, particularly in high-risk settings such as ICUs.

Our study had several limitations. Primarily, its retrospective and single-center design restricts its generalizability to other healthcare settings. Additionally, we did not systematically account for patient comorbidities or the timing of adequate antimicrobial therapy initiation, both of which are strong predictors of outcomes in KPC-CPE infections [29]. Their omission limits the conclusions that can be drawn regarding treatment efficacy. Further limitations include potential biases arising from evolving surveillance protocols, diagnostic practices, and treatment guidelines over nearly a decade, particularly those influenced by the COVID-19 pandemic. Moreover, we acknowledge that some patients classified as colonized may have been infected and vice versa, which could have introduced additional bias into our results and potentially led to the exclusion or inclusion of cases that were not accurately categorized.

Despite these limitations, our findings provide valuable insights into the complex epidemiological dynamics of KPC-CPE infections, the evolving therapeutic landscape, and the factors associated with patient mortality. This study highlights critical areas requiring immediate attention, such as targeted prevention strategies, especially in high-risk settings such as ICUs, and continued emphasis on antimicrobial stewardship practices to preserve the effectiveness of existing therapeutic options.

Future studies should investigate the underlying causes of temporal mortality fluctuations, particularly those unexplained by the available clinical data. Furthermore, prospective multicenter investigations focusing on the comparative effectiveness and safety of different antibiotic regimens are essential. These studies will be crucial for refining clinical practice guidelines and improving patient management strategies for KPC-CPE infection.

## Conclusions

Our study provides a comprehensive overview of nearly a decade of KPC-CPE infections, documenting significant fluctuations in incidence, particularly during and after the COVID-19 pandemic. The consistently high 30-day mortality rate underscores the severity of these infections, especially among critically ill patients and those with bloodstream or respiratory infections. Notably, our study documented the emergence of CZA-resistant CPE, which highlights the rapidly evolving challenge of antimicrobial resistance. Given the limited therapeutic options available, these data raise concerns about the potential for further resistance development. These findings underscore the need for sustained infection prevention efforts and rigorous antimicrobial stewardship to improve patient outcomes and effectively manage resistance risks.
